# A Universally Primed-Polymerase Chain Reaction (UP-PCR) Marker to Discriminate *Clonostachys rosea* ACM941 from Related Strains

**DOI:** 10.3390/jof5020039

**Published:** 2019-05-14

**Authors:** Zerihun A. Demissie, William G. Brown, Michele C. Loewen

**Affiliations:** 1 Aquatic and Crop Resource Development, National Research Council Canada, Ottawa, ON K1A 0R6, Canada; Zerihun.demissie@nrc.ca; 2 Adjuvants Plus Inc., 1755 Division Road N., Kingsville, ON N9Y 2Y8, Canada; bill@adjuvantsplus.com; 3 Department of Biomedical and Molecular Sciences, Queens University, Kingston, ON K7L 3N6, Canada; 4 Department of Chemistry and Biomolecular Sciences, University of Ottawa, 75 Laurier Ave E, Ottawa, ON K1N 6N5, Canada

**Keywords:** biocontrol agent, bio-fertilizer, *Clonostachys rosea*, sequence characterized amplified regions (SCAR), universal primers, UP-PCR, genetic marker

## Abstract

*Clonostachys rosea* strain ACM941 is an effective biocontrol agent against several crop diseases including Fusarium head blight. In anticipation of its increased relevance going forward, the development of a reliable DNA-based molecular marker to track it is essential. Universally primed-PCR (UP-PCR) has been used successfully to differentiate other *C. rosea* strains. Herein, the development of a UP-PCR marker for ACM941 is described. A combination of two primers (AS15 and L45) produced a ~450 bp fragment that was unique to ACM941 compared to other commercial biocontrol agents. Primers subsequently designed based on the obtained fragment also produced a similarly unique band from ACM941 alone. BLAST analysis of the amplified sequence did not yield any homologous sequence in available online databases or within the closely related *C. rosea* IK726 and CBS125111 strains’ genomes. The specificity of this marker for ACM941 was validated against ten additional *C. rosea* strains isolated from Canada, with ACM941 producing the brightest band. Taken together, these results imply that the UP-PCR primers AS15 and L45 and the amplified fragment can be used to detect and monitor the ACM941 strain after its release into the environment.

## 1. Introduction

Increasing crop productivity while taking into consideration environmental and human health concerns surrounding agricultural inputs has been a key research agenda recently [1,2]. Subsequently, identification and commercialization of beneficial microbes, including *Clonostachys rosea* f. rosea, intended for sustainable yield intensification and/or protection have grown substantially.

The genus *C. rosea* contains a number of plant endophytic and soil-borne ascomycete species, recognized for their plant development promotion property and/or protecting yield from pathogens and mycotoxin contamination. In particular, two endophytic strains isolated in North America, called 88−710 and ACM941, and the soil-borne beneficial microbe *C. rosea* f. catenulata isolate J1446 are patented as bio-stimulants and/or biocontrol agents against foliar, seed and soil-borne fungal diseases [3,4,5,6]. Strains 88−710 and J1446 are commercially available in North America as a bio-stimulant and a bio-fungicide, respectively, while commercialization of the *C. rosea* strain ACM941 to combat diverse plant pathogens, including the Fusarium head blight (FHB) causing agent *Fusarium graminearum* [7,8,9], is underway. Therefore, developing reliable identification markers to help growers or regulatory agencies distinguish these commercial strains from each other and from other related species is essential.

Morphological identification of ACM941 from other *C. rosea* strains is technically demanding and requires infrastructure not readily available. Furthermore, morphological markers cannot differentiate genetically related organisms and are culture-condition dependent. DNA-based genetic markers, on the other hand, are not affected by culture conditions or environmental factors and are both reproducible and effective for detecting intra-species variation [10]. Although several DNA-based genetic identification tools are available, the universally primed-polymerase chain reaction (UP-PCR) technique is preferred to develop strain-specific and highly reproducible markers among fungal strains. UP-PCR is a modified version of the randomly amplified polymorphic DNA (RAPD) technique where universal primers of approximately 15–21 nucleotides are used instead of the 10 nucleotide-long primers used in RAPD, which significantly enhances the specificity and reproducibility of UP-PCR-derived markers. For example, Abbasi et al. [11] screened 180 RAPD primers to identify a unique fragment differentiating *Trichoderma hamatum* strain 382 from 51 strains derived from *Trichoderma harzianum*, *Trichoderma koningii*, *Trichoderma viride* and *Trichoderma virens*. In contrast, Bulat et al. [12] screened only seven UP-PCR primers and identified a strain-specific polymorphic DNA fragment in *C. rosea* strain GR5. Paavanen-Huhtala et al. [13] also used UP-PCR to develop a strain-specific marker for isolate J1446. 

In this study, a comparative analysis of two different approaches to differentiate *C. rosea* strain ACM941 is presented. The first approach includes sequencing the highly variable regions of the nuclear ribosomal RNA (rRNA) genomic DNA called internal transcribed spacer (ITS) and D1/D2 domains. The universal rRNA comprises highly conserved multiple genes including 18S rRNA (also called small subunit (SSU)), 5.8S rRNA and 25−28S rRNA (also called large subunit (LSU)) arranged in tandem in head-to-tail fashion intervened by the highly variable ITS regions. The first 600−900 bps of the LSU gene contain the universal domains D1, D2 and D3, which are generally highly variable compared to the rest of the LSU region and often used for species identification [14]. The second approach relies on screening 45 UP-PCR primer combinations to develop a polymorphic marker distinguishing ACM941 from other biocontrol agents. In both approaches, preliminary screenings were performed on the two *C. rosea* strains of particular importance to us, ACM941 and 88−710, and only promising markers were advanced for detailed characterization. Ultimately, a UP-PCR primer-based marker that distinguishes the *C. rosea* strain ACM941 from other commercial strains was identified. The polymorphic marker was further developed into a 453 bp long sequence-characterized amplified region (SCAR) fragment that distinguishes ACM941 from other *C. rosea* strains. This PCR-based genetic fingerprinting tool will be useful to track ACM941 upon its release into the environment. 

## 2. Methods and Materials

### 2.1. Strains

Fungal strains used in the study and their respective sources are provided in Table 1.

### 2.2. Genomic Ribosomal RNA-Encoding DNA Amplification and Sequencing

Genomic DNA was extracted using EZ-10 Spin Column Fungal Genomic DNA Miniprep Kit (BioBasics, Canada). We used primers ITS1 and LR7 (Table 2) to amplify an approximately 1.5 kb region from selected *C. rosea* strains along with *T. citrinoviride* strain Tricho.12 and *T. harzianum* strain Tricho.18 (Figure S1). The two primers span the D1/D2, 5.8S, ITS-1 and ITS-2 regions. The PCR amplification was performed using 2× BesTaq master mix (ABM, Canada) in the presence of 50 ng genomic DNA and 0.1 µM of each primer in 25 µL reaction volume. The PCR program involved initial denaturation at 94 °C for 5 min, followed by 35 cycles of 30 s denaturation at 94 °C, 30 s annealing at 61 °C and 1 min extension at 72 °C, followed by a 6 min final extension cycle at 72 °C. The PCR products were cleaned using PCR clean-up kit (BioBasics, Canada) and sequenced using the primers ITS1, ITS4, NL-4, LR3R, LR0R and LR5 (Table 2), targeting different rRNA regions at National Research Council of Canada (Saskatoon). Sequence reads were analyzed and assembled using DNA Sequence Assembler v4. Partial sequences of each region for both *C. rosea* strains 88-710 and ACM941 were deposited in a gene bank database. The consensus sequences were used to retrieve homologous sequences from National Center for Biological Information (NCBI) database, aligned by ClustalW module of MEGA software and their phylogenetic relationship was deduced using its neighbor-joining phylogenetic tree construction module. Trees were visualized and annotated using the online tool in https://itol.embl.de.

### 2.3. UP-PCR Amplification and Sequencing

All nine UP-PCR primers (Table 2) were used individually and in combination with each other (with combinations forming 36 total primer sets). To identify useful primer sets out of the 45 (9 + 36) possibilities, the initial screening efforts were limited to *C. rosea* strains ACM941 and 88-710 genomic DNA alone and only polymorphic primers were advanced for full-scale characterization work. The UP-PCR was performed in triplicate using 2× BesTaq master mix (ABM, Canada) in the presence of 70 ng genomic DNA and 0.2 µM final primer concentration in 20 µL reaction volume. The PCR program involved initial denaturation at 94 °C for 7 min, followed by 35 cycles of 50 s denaturation at 94 °C, 70 s annealing at 55 °C and 60 s extension at 72 °C, followed by a 3 min final extension cycle at 72 °C. The ramping rate was about 2.4 °C/s. Half of the reaction volume (10 µL) from each replicate was resolved on 7.5 cm long 6% polyacrylamide gel at 160 V for 55 min using Tris-Borate-EDTA running buffer and stained with SYBR-safe (Thermo Fisher Scientific, Canada). The bands were visualized and documented using the SYBR-safe module of BioRad Chemidoc imaging system (BioRad, Canada).

The SDS-PAGE slices containing the two unique bands from the ACM941 and 88-710 strains were excised and the DNA was isolated following a combination of the ‘crush and soak’ method [21] and DNA ethanol precipitation. Briefly, the excised fragments were chopped into fine pieces using razor blades and soaked overnight at 37 °C on a slow rotating platform in 1 volume of elution buffer (0.5 M ammonium acetate and 1 mM EDTA, pH 8.0). The sample was then spun at 10,000 g for 10 min to pellet gel slices and the supernatant was transferred into clean tubes. An additional 0.5 volume of elution buffer was added to the pellet, vortexed vigorously and the supernatant was recovered as described earlier. The two supernatants were combined and the DNA was precipitated by an ethanol precipitation method by adding 2 volumes of 100% ethanol. The mix was incubated at −20 °C for 30 min and spun at 12,000 g for 12 min at 4 C to pellet DNA. The supernatant was removed gently and the pellet was washed in 200 µL of ice-cold 70% ethanol, followed by centrifugation at 12,000 *g* for 3 min at 4 °C. The supernatant was discarded and the pellet was air-dried with the DNA re-suspended in TE buffer. The fragments were cloned into pGEM-T-Easy vectors following the manufacturer’s guidelines (Promega, USA) and sequenced. The sequences were mapped against *C. rosea* strains IK726 [22] and CBS125111 (https://genome.jgi.doe.gov/Cloro1/Cloro1.home.html) genome sequences using BLASTn and subjected to a homology search against the NCBI nucleotide nr database to determine their uniqueness. The ACM941 fragment was selected for further analysis based on the homology search result.

### 2.4. ACM941 SCAR Specific Probe Development and Southern Blotting

The ACM941 SCAR specific DIG-labelled DNA probes were generated using the DIG High Prime DNA Labeling and Detection Starter Kit I (Roche Diagnostics, Indianapolis, USA) following the manufacturer’s guidelines from PCR-amplified fragments using vectors harboring the cloned fragment as a template in the presence of Phusion^®^ High-Fidelity DNA Polymerase (NEB, Canada) and 0.1 µM each of ACM941-F and ACM941-R primers (Table 2). The PCR program involved 98 °C for 30 s, followed by 20 cycles of 10 s denaturation at 98 °C, 30 s annealing at 62 °C and 30 s extension at 72 °C, followed by a 5 min final extension cycle at 72 °C. Then, we performed PCR using 50 ng genomic DNA of different *C. rosea* strains and 2× BesTaq master mix (ABM, Canada) in the presence of 0.2 µM of individual primers of either As15/L21 or ACM941-F/ACM941-R (Table 1) combinations in 20 µL reaction volume. The PCR program was initial denaturation at 94 °C for 7 min, followed by 35 cycles of 50 s denaturation at 94 °C, 70 s annealing at 55 °C or 62 °C, respectively, and 60 s extension at 72 °C, followed by a 3 min final extension cycle at 72 °C. The ramping rate was about 2.4 °C/s. Following this, 2 µL of each PCR product, along with 5 µg of EcoRI-digested SCAR fragment containing the pGEM-T-Easy vector, were resolved on 1.5% agarose gel. The procedure outlined in the DIG High Prime DNA Labeling and Detection Starter Kit I (Roche Diagnostics, Indianapolis, IN, USA) manual was followed for DNA transfer to a nylon membrane, fixation, hybridization and immunological detection. Briefly, the blot result was photographed using the colorimetric module in the BioRad Chemidoc imaging system (BioRad, Canada).

## 3. Results

### 3.1. Nuclear Encoded Ribosomal RNA Gene Amplification and Analysis

The variable domain D1/D2 partial sequences amplified from the *C. rosea* strains ACM941 and 88-710 were 796 and 756 bp long, respectively, while those of ITS-1 and ITS-2 spanning the 5.8S regions were 501 and 452 bp long, respectively (Table 3). The difference in D1/D2 and ITS sizes of the two strains was due to sequencing result quality differences. However, the overlapping regions of each region from both strains were identical. Although not sequenced, the PCR fragments amplified using the ITS1 and LR5 primer combination from the two *C. rosea* strains DAOM175083 and DAOM214828 were of similar sizes to those of *C. rosea* strains ACM941 and 88-710, while the fragment amplified from *T. harzianum* strain Tricho.18 had a lower molecular weight. The fragment from *C. rosea* isolate J1446 was slightly smaller than that of *C. rosea* strains (Figure S1A). With respect to the ITS1 specific primers ITS1-X and ITS1-Y (Table 2) described by Bulat et al. [17], similar molecular weight fragments were obtained from the above four *C. rosea* strains. However, the molecular weight of fragments amplified from Tricho.18 and Tricho.12 were different from each other and also from that of *C. rosea* strains, while a faint band with a molecular size similar to that of the *C. rosea* bands was obtained from J1446 (Figure S1B). The apparent high degree of rRNA sequence identity observed here among *C. rosea* strains is not unexpected, even with respect to strains originating from different geographic locations. For example, D1/D2 regions amplified from eight *S. boulardii* strains showed 100% sequence identity [23]. In line with this, the D1/D2 region of *C. rosea* strains ACM941 and 88-710 displayed a 100% nucleotide sequence identity with *C. rosea* strains CBS 128894, CBS 127294, CBS 125.72, CBS 443.65, CBS 277.50, CBS 226.48, PAV-M 1.025, G3-1, *C. rosea* culture-collection MUT<ITA>:4900 and *Bionectria ochroleuca* strain CCFC226708. Furthermore, BLAST analysis of both D1/D2 and ITS-1/ITS2 region sequences against NCBI nr database retrieved an additional 58 fungal strains with 99% sequence identity, suggesting a high level of rRNA genetic sequence conservation in fungal species in general (Figure S2a,b).

Contrary to the partial LSU region, only five isolates in four species, including *Trichoderma* sp. isolate yi1252_1, *C. rosea* strain pC1, *B. ochroleuca* strain ATCC 48395, *B. ochroleuca* isolate SB_Bac6 and *Nectria gliocladioides* strain S9A6, showed 100% similarity to the 5.8S and partial ITS regions of both ACM941 and 88-710. However, out of the 100 homologues retrieved from the NCBI nr database using the partial D1/D2 region as a query a maximum of three nucleotide substitutions (99% identity) were observed. In comparison, blasting the partial LSU (D1/D2) region of ACM941 against NCBI nr database retrieved homologues containing up to 29 bp differences/96% sequence identity (data not shown). We also tried to amplify the cpn60-type molecular chaperone’s genomic DNA, however, repeated attempts did not produce any distinct band from either strain (data not shown). 

### 3.2. UP-PCR Markers Distinguishing C. rosea Strain ACM941 from 88-710

**Figure 1:**
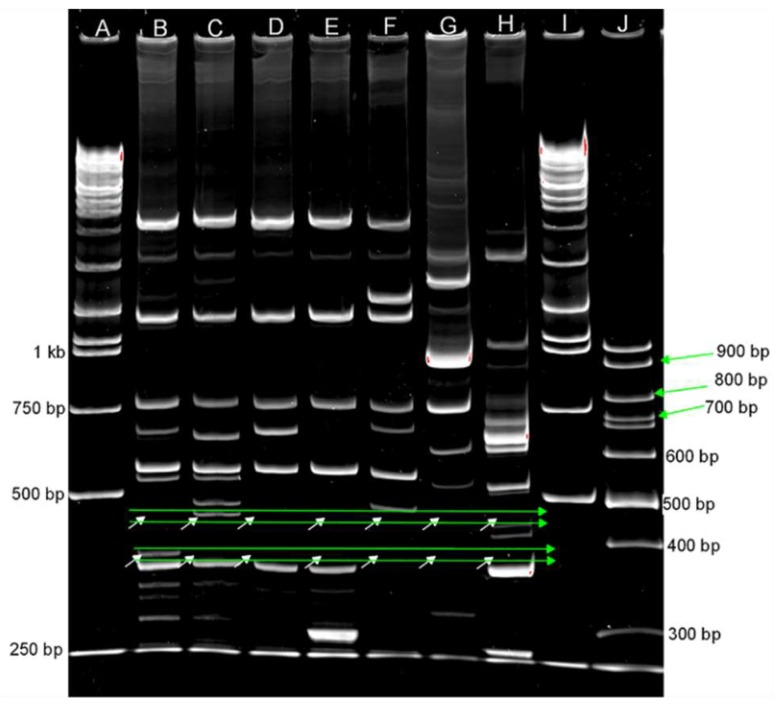
PCR products of *C. rosea* isolates using the AS15 and L21 primer combination resolved on 6% polyacrylamide gel. Lane descriptions: **A**—ExcelBand™ XL 25 kb DNA Ladder, Broad Range; **B**—*C. rosea* strain 88-710; **C**—*C. rosea* strain ACM941; **D**—*C. rosea* strain DAOMC175083; **E**—*C. rosea* strain DAOMC241828; **F**—*C. rosea* isolate J1446; **G**—*T. citrinoviride* strain Tricho.12; **H**—*T. harzianum* strain Tricho.18; **I**—ExcelBand™ XL 25 kb DNA Ladder, Broad Range; and **J**—NEB 100 bp DNA Ladder. White arrows indicate expected band positions of the unique fragments.

Of the 45 possible primer combinations tested (Table 2), only nine produced distinct bands differentiating the *C. rosea* strain ACM941 from 88-710 and were advanced for further analysis. The primer combinations were: As15 + L45, As15 + l21, As15inv + L21, As15 + AA2M2, As4 + L45, As15inv + Fok1a, As15 + 3-2, As15inv + AA2M2 and L21 alone. Of these, only the AS15 + L45 primer combination produced a ~450 bp fragment that was specific to ACM941 and a ~370 bp that initially appeared to be specific for strain 88-710 (Figure 1). These results were validated by repeating the PCR reactions on two additional separate genomic DNA extractions, yielding consistent results. Sequencing the ACM941 specific fragment, hereafter referred to as SCAR-2, revealed a 453 bp long sequence (accession number: MK650463) with no homology to any genome sequences of *C. rosea* strains IK726 and CB125111 or to any sequence in the NCBI database as of the time of writing (Table S1). On the other hand, sequencing of the fragment amplified from strain 88-710, referred to as SCAR-3, revealed a 404 bp-long sequence that was successfully mapped to scafold_547 of *C. rosea* strain IK726 with 97% identity and coverage) showing significant homology to a serine/threonine-protein kinase both at the nucleotide and translated protein level by BLAST against the NCBI database (Table S1). Subsequent investigation using *de novo* SCAR primers designed on the basis of the obtained SCAR-3 sequence also amplified a fragment of similar size from strain ACM941 (data not shown). Therefore, SCAR-3 was omitted from further analysis. Two additional fragments (SCAR-1 and SCAR-4) were also sequenced (Figure 1) and both were mapped to both *C. rosea* genomes (Table S1).

**Figure 2:**
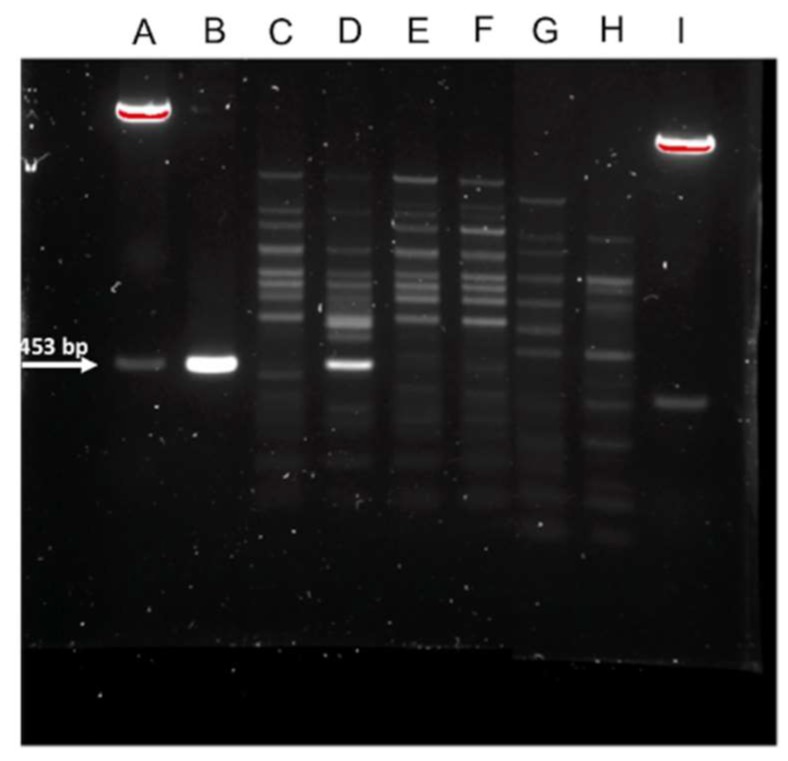
SCAR-2 primer amplification products resolved on 1.5% agarose gel. Lane descriptions: **A**—EcoRI-digested pGEM-T vector harboring SCAR-2; **B—**PCR-amplified SCAR-2; **C**—*C. rosea* strain 88-710; **D**—*C. rosea* strain ACM941; **E**—*C. rosea* isolate J1446; **F**—*C. rosea* strain DAOMC241828; **G**—*C. rosea* strain DAOMC175083; **H**—*C. rosea* strain DAOMC238388; and **I**—EcoRI-digested pGEM-T vector harboring SCAR-3.

**Figure 3:**
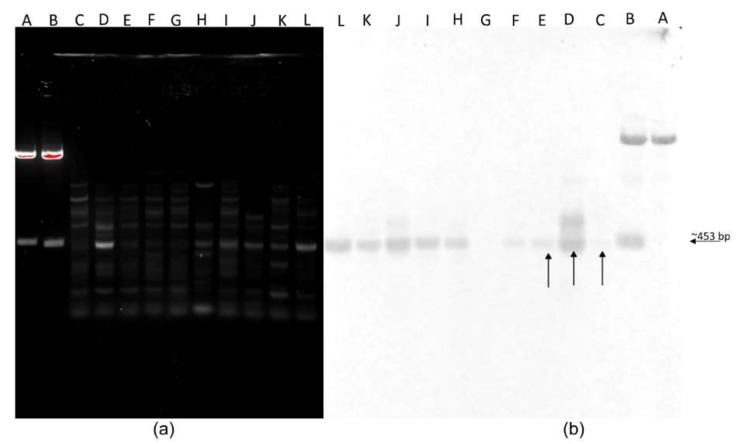
SCAR-2 primer products detection using gel electrophoresis and Southern blot. (**a**) SCAR-2 primer amplification products resolved on 1.5% agarose gel and (**b**) Southern blot results using DIG-labelled ACM941 specific SCAR probe. Lane descriptions: **A**—plasmid-24 RE (negative control); **B**—plasmid-20 RE (positive control); **C**—*C. rosea* strain 88-710; **D**—*C. rosea* strain ACM941; **E**—*C. rosea* strain J1446; **F**—*C. rosea* strain DAOMC241828; **G**—*C. rosea* strain DAOMC175083; **H**—*C. rosea* strain DAOMC250196; **I**—*C. rosea* strain DAOMC251432; **J**—*C. rosea* DAOMC238301; **K**—*C. rosea* strain DAOMC238388; and **L**—*C. rosea* strain DAOMC226796. Labels on the gel are given in bold letters. Patented *C. rosea* strains are indicated by arrows.

Interestingly, SCAR primers (Table 2) designed against SCAR-2 produced a strong band from *C. rosea* ACM941 but not from the two other commercial biocontrol agent *C. rosea* strains 88-710 or J1446, nor *C. rosea* isolates DAOM175083 and DAOM214828 (Figure 2). Encouraged by this specificity, additional available strains were tested. Here, faint bands of similar molecular weight were detected from *C. rosea* isolates DAOMC250196, DAOMC251432, DAOMC238301, DAOMC226796 and DAOMC238388 (Figure 3a). Similarly, Southern blot assays against PCR-amplified genomic DNA products using DIG-labelled ACM941 SCAR-2 failed to detect the sequence in either of the other two commercial strains or the two *C. rosea* isolates tested earlier, but yielded faint bands in all other strains. The non-specificity of the DIG-labelled probes was also evident from cross-reaction with the pGEM-T Easy vector backbone (Figure 3b), although there was no significant homology between the probe and the vector.

## 4. Discussion

From the discovery of penicillin to the recently identified new class of antibiotic teixobactin [24] and emergence of microbiome engineering, humans have, and continue to, harness the potential of fungal organisms. With the world population expected to surpass nine billion by the year 2050 and environmental and human health concerns associated with agricultural inputs steadily growing, the prospect of taking advantage of plant growth and yield protection properties of beneficial fungi, including *C. rosea,* cannot be ignored. The UP-PCR marker and SCAR primers derived from this study show a promising effectiveness in distinguishing *C. rosea* strain ACM941 from 88-710 and also from other morphologically similar biocontrol agents like *C. rosea* strain IK726 [22] and J1446 [6].

Amplification of rRNA regions, particularly the inter-transcribed regions (ITS1 and ITS2), 5.8S and the highly variable D1/D2 region, are commonly used in fungal systematic studies [25]. However, high levels of sequence conservation in these regions is also a common phenomenon, especially between intra-species organisms. For example, Bulat et al. [17] reported ribotyping of strains from *Trichoderma* and *C. rosea* spp. into separate ITS groups, but no difference was observed among strains belonging to each group. Paavanen-Huhtala et al. [13] also reported that the 28S, ITS1-5.8S-ITS2 and 18S regions amplified from 17 *C. rosea* strains, including J1446, were found to share identical sequences. Similarly, the partial D1/D2 and ITS1-5.8S-ITS2 regions amplified from *C. rosea* strain ACM941 and 88-710 in this work were 100% identical. In addition, nine *C. rosea* strains retrieved from public databases and *B. ochroleuca* strain CCFC226708 shared 100% sequence identity to D1/D2 region of ACM941 and 88-710. On the other hand, *C. rosea* strain pC1 and four other fungal species, *Trichoderma* sp. isolate yi1252, *B. ochroleuca* strain ATCC 48395, *B. ochroleuca* isolate SB_Bac6 and *N. gliocladioides*, showed 100% sequence identity against the ITS1-5.8S-ITS2 region of ACM941 and 88-710. 

It is also worth noting that, while the D1/D2 ACM941 sequence top alignment hit list is dominated by *C. rosea* strains, only one *C. rosea* strain showed 100% conservation against ITS1-5.8S-ITS2 sequences. Of the nine *C. rosea* strains with a 100% conserved D1/D2 region to ACM941/88-710 strains, four of them had a 1–2 bp difference in their ITS1-5.8S-ITS2 regions. On the other hand, none of the fungal species that showed 100% sequence identity with the ITS1-5.8S-ITS2 region of ACM941 were found in the top 100 alignment results against the D1/D2 region (data not shown). Although this might imply a high degree of ITS1-5.8S-ITS2 region conservation across fungal species, the molecular weight of the *T. citrinoviride* strain Tricho.12 and the *T. harzianum* strain Tricho.18 ITS1 region amplified using ITS1-X and ITS1-Y primers (Table 1) showed significant deviation from the five *C. rosea* strains tested in this study (Figure S1B). This discrepancy is likely because only partial ITS1 sequences of the *C. rosea* strain ACM941 were obtained, such that the size differences do not necessarily contradict the alignment or the phylogenetic tree deduced from it (Figure S2a,b). 

In contrast to rRNA genomic regions, UP-PCR primers generally do not show homology to sequences in public database and have been used to develop strain-specific markers for *C. rosea* strain GR5 [12,26]. Similarly, Lubeck et al. [19] used UP-PCR primers to precisely assign several *R. solani* isolates into their correct anastomosis subgroups. In this work, nine UP-PCR primers (Table 2) were tested in order to develop a specific marker to distinguish *C. rosea* strain ACM941 from other patented *C. rosea* strains and known biocontrol agents, such as *T. citrinoviride* and *T. harzianum*. The combination of the UP-PCR primers AS15 and L45 produced a 453 bp-long unique fragment in strain ACM941 (Figure 1) and the SCAR primers derived from the unique SCAR-2 fragment also produced an unambiguous band specific to strain ACM941 compared to all other patented biocontrol agents (Figure 2). 

The intent of this project was to develop a unique and reproducible molecular marker to differentiate ACM941 and 88-710 from each other and similar biocontrol agents. Two specific fragments each specific for 88-710 and ACM941 were initially obtained using AS15 and L45 primers (Figure 1). However, the *C. rosea* strain 88-710 unique fragment (SCAR-3, Table S2) was detected in ACM941 during more in-depth analysis (data not shown) and thus was not considered in further screening. SCAR-2, on the other hand, efficiently discriminated ACM941 from all commercial biocontrol agents, including *C. rosea* strains 88-710 and J1446 (Figure 2 and Figure 3a,b). The SCAR-2 sequence did not show homology to any other known sequence, including the closely related *C. rosea* strain IK726 genome. This was despite the fact that IK726 sequences were used previously to design cloning primers for genes encoded in strain ACM941 and previous transcriptomic analyses showed that the two strains share very high sequence identity [27]. Of the four fragments we cloned (Figure 1), only SCAR-2 did not identify a homologous region in the IK726 genomic sequence (Table S1). SCAR-2 primers (Table 2) also either failed to detect a visible band or produced only a very faint band in eight other *C. rosea* strains (Figure 3a). The relatively low intensities of these bands are indicative of their low homology to SCAR-2. The DIG-labelled probe derived from SCAR-2, however, yielded higher intensity bands for strains other than ACM941. This is likely due the inherent non-specificity associated with DIG-labelled probes. Indeed, even the best optimized conditions still allow DIG-labelled probes to bind to sequences with as low as 80%–85% identity (DIG Manual, Roche Diagnostics, Indianapolis, USA). Similarly, and perhaps going to the robustness of the UP-PCR SCAR-2 primers developed here, previously identified UP-PCR markers were found to detect bands with high sequence homology from some other strains. For example, the *C. rosea* strain GR5-specific marker developed by Bulat et al. [12] was found to detect fragments with 96%−99% sequence similarity from some strains isolated from different geographic locations. Therefore, taken together, these results imply that the SCAR-2 sequence is likely unique to ACM941 and can be used to detect and monitor strain ACM941 after its release to the environment.

## 5. Conclusions

In conclusion, we have developed a PCR-based genetic fingerprinting method that can distinguish the *C. rosea* strain ACM941 from other *C. rosea* strains and related biocontrol agents in use in North America. *C. rosea* strains ACM941 and 88-710 and other similar patented strains share a high degree of application overlap (crops), although they are intended for different purposes. Therefore, distinguishing these strains before application (manufacturing and handling stages) to maintain their purity is as important as tracking them after their application. The marker described here can efficiently characterize ACM941 from these strains. Efforts are underway to test the effectiveness of these loci in identifying ACM941 from natural populations. Once the protocol is optimized, the primers can be used to track ACM941 after its release. 

## Figures and Tables

**Table 1:** Fungal strains used in this study.

Genus	Strain/Isolate	Source
*C. rosea*	ACM941	Adjuvant Plus Inc. (Kingsville, ON)
*C. rosea*	88-710	Adjuvant Plus Inc. (Kingsville, ON)
*C. rosea*	J1446	Adjuvant Plus Inc. (Kingsville, ON)
*C. rosea*	DAOM175083	Canadian Collection of Fungal Cultures (DAOMC) (Ottawa, Canada)
*C. rosea*	DAOMC214828	Canadian Collection of Fungal Cultures (DAOMC) (Ottawa, Canada)
*C. rosea*	DAOMC174998	Canadian Collection of Fungal Cultures (DAOMC) (Ottawa, Canada)
*C. rosea*	DAOMC144742	Canadian Collection of Fungal Cultures (DAOMC) (Ottawa, Canada)
*C. rosea*	DAOMC186891	Canadian Collection of Fungal Cultures (DAOMC) (Ottawa, Canada)
*C. rosea*	DAOMC39046	Canadian Collection of Fungal Cultures (DAOMC) (Ottawa, Canada)
*C. rosea*	DAOMC250202	Canadian Collection of Fungal Cultures (DAOMC) (Ottawa, Canada)
*C. rosea*	DAOMC250197	Canadian Collection of Fungal Cultures (DAOMC) (Ottawa, Canada)
*C. rosea*	DAOMC250196	Canadian Collection of Fungal Cultures (DAOMC) (Ottawa, Canada)
*C. rosea*	DAOMC226796	Canadian Collection of Fungal Cultures (DAOMC) (Ottawa, Canada)
*C. rosea*	DAOMC251432	Canadian Collection of Fungal Cultures (DAOMC) (Ottawa, Canada)
*C. rosea*	DAOMC238388	Canadian Collection of Fungal Cultures (DAOMC) (Ottawa, Canada)
*C. rosea*	DAOMC238301	Canadian Collection of Fungal Cultures (DAOMC) (Ottawa, Canada)
*C. rosea*	DAOM175083	Allen Xue’s Lab—Ottawa Center for Research and Development (ON)
*C. rosea*	DAOM214828	Allen Xue’s Lab—Ottawa Center for Research and Development (ON)
*T. citrinoviride*	Tricho.12	Allen Xue’s Lab—Ottawa Center for Research and Development (ON)
*T. harzianum*	Tricho.18	Allen Xue’s Lab—Ottawa Center for Research and Development (ON)

**Table 2:** Primers used in this study.

Primer	Sequence (5’ to 3’)	Length (bp)	Reference
rRNA			
ITS1	TCCGTAGGTGAACCTGCGG	19	[15]
ITS4	TCCTCCGCTTATTGATATGC	20	[15]
LR7	TACTACCACCAAGATCT	17	[16]
NL-4	GGTCCGTGTTTCAAGACGG	19	[16]
LR0R	ACCCGCTGAACTTAAGC	17	[16]
LR5	TCCTGAGGGAAACTTCG	17	[16]
LR3R	GTCTTGAAACACGGACC	17	[16]
ITS1-X	TGAACCTGCGGAAGGATCATT	21	[17]
ITS1-Y	GCATTTCGCTGCGTTCTTCAT	21	[17]
UP-PCR			
AS4	TGTGGGCGCTCGACAC	16	[12]
AS15	GGCTAAGCGGTCGTTAC	17	[12]
AS15inv	CATTGCTGGCGAATCGG	17	[12]
AA2M2	CTGCGACCCAGAGCGG	16	[12]
Fok1a	GGATGACCCACCTCCTAC	18	[12]
L21	GGATCCGAGGGTGGCGGTTCT	21	[12]
M	TAAGGGCGGTGCCAGT	16	[12]
L45	GTAAAACGACGGCCAGT	17	[18]
L15/AS19	GAGGGTGGCGGCTAG	15	[19]
SCAR-2			
ACM941-F	TGGATCCGAGGGTGGCGGTTCTA	23	This study
ACM941-R	TGGCTAAGCGGTCGTTACTACCAAAGATCC	30	This study
Cpn60			
M13F40	GAIIIIGCIGGIGAYGGIACIACIAC/YKIYKITCICCRAAICCIGGIGCYTT		[20]
M13r48	GAIIIIGCIGGYGACGGYACSACSAC/CGRCGRTCRCCGAAGCCSGGIGCCTT		[20]

**Table 3:** Accession number of rRNA genomic regions amplified from *C. rosea* strains ACM941 and 88-710.

Accession Number	Strain	rRNA Region	Sequencing Primers
MK391601	ACM941	Partial D1/D2 region; 28S ribosomal RNA gene partial sequence	NL-4, LR3R, LR0R, LR5
MK391602	88-710	Partial D1/D2 region; 28S ribosomal RNA gene partial sequence	NL-4, LR3R, LR0R, LR5
MK411420	ACM941	ITS-1 and ITS-2 partial and 5.8S complete rRNA sequences	ITS1, ITS4
MK411421	88-710	ITS-1 and ITS-2 partial and 5.8S complete rRNA sequences	ITS1, ITS4

## Supplementary Materials

The following are available online at https://www.mdpi.com/2309-608X/5/2/39/s1. PDF document entitled Supplementary Tables and Figures, which includes Table S1, Figures S1 and S2.
