# Supplementary material for: A Universally Primed-Polymerase Chain Reaction (UP-PCR) Marker to Discriminate Clonostachys rosea ACM941 from Related Strains
Source: J Fungi (Basel). 2019 May 14;5(2):39. doi: 10.3390/jof5020039 (PMC6617100; doi:10.3390/jof5020039)
Supplement: Supplementary file 1 [file jof-05-00039-s001.pdf]

A Universally Primed-PCR (UP-PCR) marker to discriminate *Clonostachys rosea* ACM941 from related strains

Zerihun A. Demissie[1], William G. Brown[2] and Michele C. Loewen[1,3,4*]

[1]Aquatic and Crop Resource Development, National Research Council Canada, Ottawa, ON, Canada; Zerihun.demissie@nrc.ca
[2]Adjuvants Plus Inc. 1755 Division Road N., Kingsville, ON Canada; bill@adjuvantsplus.com
[3]Department of Biomedical and Molecular Sciences, Queens University, Kingston, ON, Canada; michele.loewen@nrc.ca
[4]Department of Chemistry and Biomolecular Sciences, University of Ottawa, 75 Laurier Ave E, Ottawa, ON K1N 6N5
*Corresponding author: michele.loewen@nrc.ca

Supplementary Tables and Figures

		BLASTn analysis against				
		IK726 genome		CBS125111 genome		NCBI nucleotide database
ID	Size (bp)		Location		Location	
SCAR-1	466	+ve	scf_003: 1010111 - 1010558	+ve	Scaffold-1:2996275-2996690	-ve
**SCAR-2**	**453**	**-ve**	**N/A**	**-ve**	**N/A**	**-ve**
SCAR-3	404	+ve	scf_547: 1756-2148	-ve	N/A	+ve
SCAR-4	375	+ve	scf_033: 132172-132536	+ve	Scaffold-14:1082620-1082973	-ve

Supplementary Table 1. BLASTn analysis of the four SCAR fragments cloned and sequenced. SCAR-1 is the higher molecular weight fragment from strain ACM941 (boxed in Figure 1), SCAR-2 is the lower molecular weight fragment from strain ACM941 (boxed in Figure 1), SCAR-3 is the higher molecular weight fragment from strain 88-710 (boxed in Figure 1) and SCAR-4 is the lower molecular weight fragment from strain 88-710 (boxed in Figure 1).

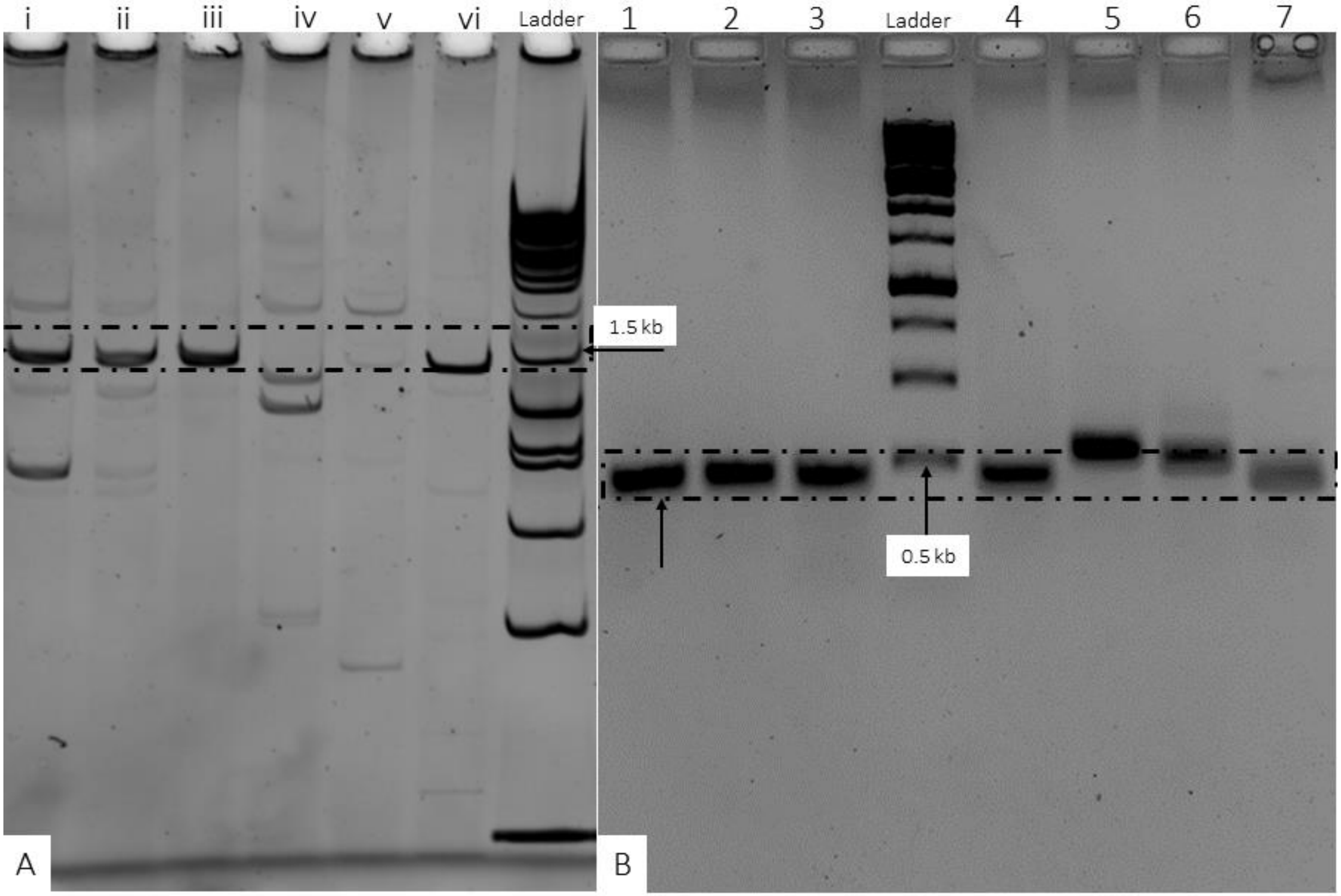

Supplementary Figure 1. Amplification of rRNA regions from *C. rosea* strains and other biocontrol fungi. (a) PCR products of ITS1 + LR7 primers resolved on 6% SDS-PAGE and (b) PCR products of ITS1-X + ITS1-Y primers resolved on 1.5% agarose gel. Label descriptions: **i** - *C. rosea* strain 88-710; **ii** - *C. rosea* strain ACM941; **iii** - *C. rosea* strain DAOMC175083; **iv** – *T. citrinoviride* strain Tricho.12; ***v*** – *T. harzianum* strain Tricho.18; **vi** - *G. Catenulatum* strain J1446*;* **1** - *C. rosea* strain 88-710; **2** - *C. rosea* strain ACM941; **3** - *C. rosea* strain DAOMC175083; **4** - *C. rosea* strain DAOMC241828; **5** – *T. citrinoviride* strain Tricho.12; ***6*** – *T. harzianum* strain Tricho.18 *and* **7** - *G. Catenulatum* strain J1446*.*

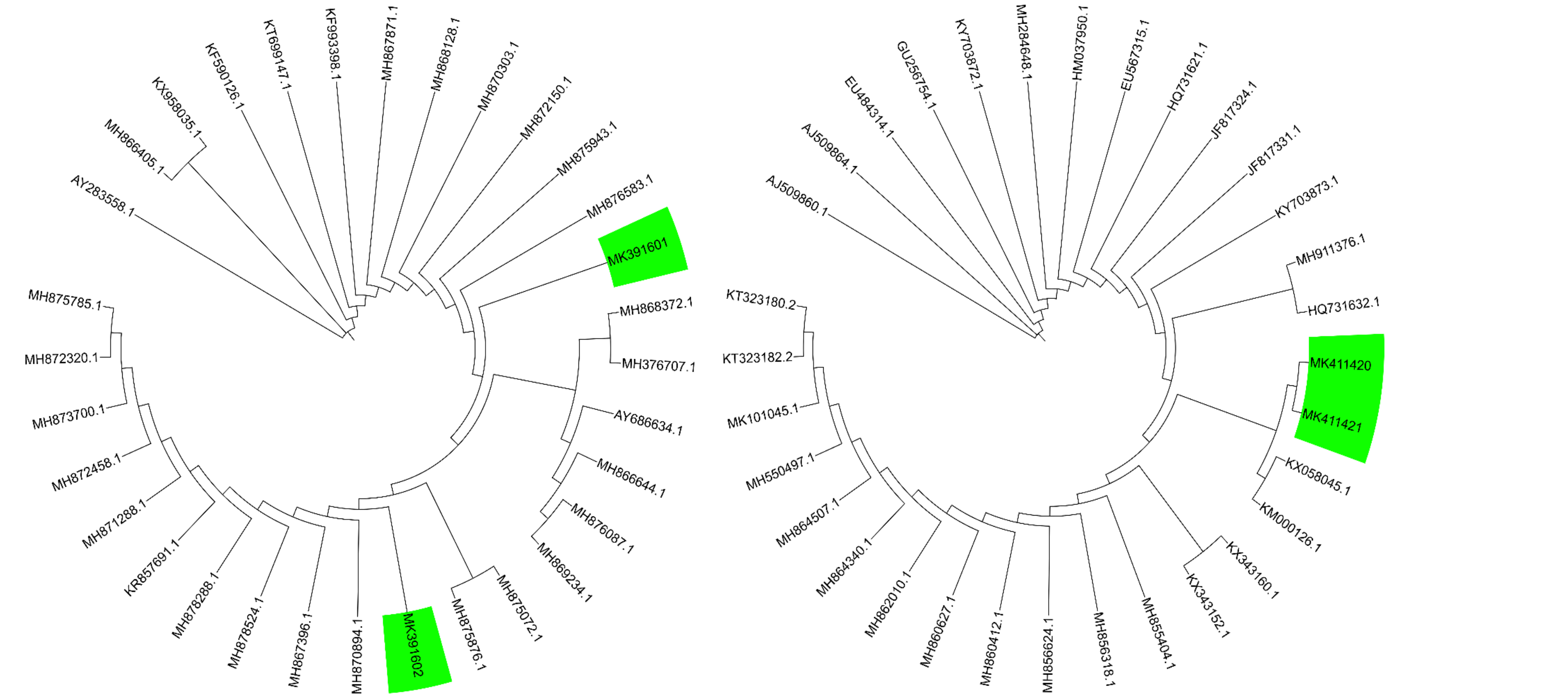

Supplementary Figure 2. Phylogenetic relationships among different C. rosea strains and other selected species based on (a) partial D1/D2 region and (b) Partial ITS-1and ITS2 and complete 5.8S regions.

Accession number descriptions of A (arranged based on their similarity to C. rosea strain ACM941 D1/D2 region from high to low): MK391601 C. rosea strain_ACM941 partial D1/D2 region; MK391602 - C. rosea strain_88-710 partial D1/D2 region; MH878524.1 - C. rosea strain CBS 907.72F large subunit ribosomal RNA gene; MH876583.1 - C. rosea strain CBS 128894 large subunit ribosomal RNA gene; MH875943.1 - C. rosea strain CBS 127294 large subunit ribosomal RNA gene; MH875785.1 - C. rosea strain CBS 126933 large subunit ribosomal RNA gene; MH873700.1 - C. rosea strain CBS 710.86 large subunit ribosomal RNA gene; MH872458.1 - C. solani f. nigrovirens strain CBS 469.73 large subunit ribosomal RNA gene; MH872320.1 - C. rosea strain CBS 907.72B large subunit ribosomal RNA gene; MH872150.1 - C. rosea f. catenulata strain CBS 125.72 large subunit ribosomal RNA gene; MH871288.1 - G. aureum strain CBS 998.69 large subunit ribosomal RNA gene; MH870303.1 - C. rosea f. catenulata strain CBS 443.65 large subunit ribosomal RNA gene; MH868128.1 - C. rosea strain CBS 277.50 large subunit ribosomal RNA gene; MH867871.1 - C. rosea strain CBS 226.48 large subunit ribosomal RNA gene; KR857691.1 - C. rosea strain CBS 710.86 28S ribosomal RNA gene; KF993398.1 - C. rosea isolate PAV-M 1.025 28S ribosomal RNA gene; KT699147.1 - C. rosea culture-collection MUT<ITA>:4900 28S ribosomal RNA gene; KF590126.1 - Gliocladium sp. G3-1 28S ribosomal RNA gene; AY283558.1 - B. ochroleuca strain CCFC226708 large subunit ribosomal RNA gene; MH878288.1 - C. rosea strain CBS 906.72A large subunit ribosomal RNA gene; MH876087.1 - C. rosea strain CBS 127642 large subunit ribosomal RNA gene; MH875876.1 - C. rosea strain CBS 127143 large subunit ribosomal RNA gene; MH870894.1 - C. rosea strain CBS 438.68 large subunit ribosomal RNA gene; MH869234.1 - C. rosea strain CBS 194.57 large subunit ribosomal RNA gene; MH867396.1 - C. rhizophaga strain CBS 202.37 large subunit ribosomal RNA gene; MH866644.1 - N. camerunensis strain CBS 225.31 large subunit ribosomal RNA gene; MH866405.1 - C. rosea f. catenulata strain CBS 154.27 large subunit ribosomal RNA gene; MH875072.1 - C. rhizophaga strain CBS 125416 large subunit ribosomal RNA gene; AY686634.1 - B. ochroleuca 28S ribosomal RNA gene; MH376707.1 - C. krabiensis voucher MFLUCC 16-0254 28S large subunit ribosomal RNA gene; MH868372.1 - C. rosea strain CBS 271.51 large subunit ribosomal RNA gene; and KX958035.1 - C. rosea isolate 26R-8-F03 internal transcribed spacer 1. Accession number descriptions of B (arranged based on their similarity to C. rosea strain ACM941 ITS-5.8S-ITS region from high to low): MK41142 - C. rosea strain_ACM941 ITS-1 and ITS-2 partial and 5.8S complete rRNA sequences; MK411421 - C. rosea strain_88-710 ITS-1 and ITS-2 partial and 5.8S complete rRNA sequences; MH284648.1 - Trichoderma sp. isolate yi1252_1 small subunit ribosomal RNA gene; KY703872.1 - C. rosea strain pC1 18S ribosomal RNA gene; GU256754.1 - B. ochroleuca strain ATCC 48395 18S ribosomal RNA gene, partial sequence; EU484314.1 - B. ochroleuca isolate SB_Bac6 18S ribosomal RNA gene, partial sequence; AJ509864.1 - Nectria gliocladioides 5.8S rRNA gene; KY703873.1 - C. rosea strain pP3 18S ribosomal RNA gene; KX343160.1 - Clonostachys sp. isolate GETM11 18S ribosomal RNA gene, partial sequence; internal transcribed spacer 1; KX343152.1 - C. rosea isolate 22PDA-4C2 18S ribosomal RNA gene; KX058045.1 - C. rosea strain qlz6 18S ribosomal RNA gene; KM000126.1 - Fungal sp. BVK-SMA-2 18S ribosomal RNA gen; JF817331.1 - C. rosea strain CanS-56 18S ribosomal RNA gene; JF817324.1 - C. rosea strain CanS-48 18S ribosomal RNA gene; HQ731632.1 - Bionectria sp. Papochf 04 18S ribosomal RNA gene; HQ731621.1 - Bionectria sp. Macof 02 18S ribosomal RNA gene; HM037950.1 - B. ochroleuca strain wxm110 18S ribosomal RNA gene; EU567315.1 - B. ochroleuca strain EXGF-1 18S ribosomal RNA gene; AJ509860.1 - N. gliocladioides 5.8S rRNA gene; KT323182.2 - C. rosea isolate HP113 18S ribosomal RNA gene; KT323180.2 - C. rosea isolate HP111 18S ribosomal RNA gene; MK101045.1 - C. rosea isolate UCR3250 small subunit ribosomal RNA gene; MH550497.1 - Clonostachys sp. strain daef27 small subunit ribosomal RNA gene; MH911376.1 - C. rosea strain MF22417 small subunit ribosomal RNA gene; MH864507.1 - C. rosea strain CBS 127294 small subunit ribosomal RNA gene; MH864340.1 - C. rosea strain CBS 126933 small subunit ribosomal RNA gene; MH862010.1 - C. rosea strain CBS 710.86 small subunit ribosomal RNA gene; MH860627.1 - C. rosea strain CBS 907.72B small subunit ribosomal RNA gene; MH860412.1 - C. rosea f. catenulata strain CBS 125.72 small subunit ribosomal RNA gene; MH856624.1 - C. rosea strain CBS 277.50 small subunit ribosomal RNA gene; MH856318.1 - C. rosea strain CBS 226.48 small subunit ribosomal RNA
